# Direct detection of brown adipose tissue thermogenesis in UCP1−/− mice by hyperpolarized ^129^Xe MR thermometry

**DOI:** 10.1038/s41598-019-51483-4

**Published:** 2019-10-16

**Authors:** Michael A. Antonacci, Christian McHugh, Michele Kelley, Andrew McCallister, Simone Degan, Rosa T. Branca

**Affiliations:** 1 Department of Physics and Astronomy, University of North Carolina at Chapel Hill, Chapel Hill, North Carolina United States of America; 2 Biomedical Research Imaging Center, University of North Carolina at Chapel Hill, Chapel Hill, North Carolina United States of America; 3 Department of Radiology, Duke University, Durham, North Carolina United States of America; 4 Present Address: Department of Physics, Saint Vincent College, Latrobe, Pennsylvania United States of America

**Keywords:** Preclinical research, Magnetic resonance imaging, Endocrinology

## Abstract

Brown adipose tissue (BAT) is a type of fat specialized in non-shivering thermogenesis. While non-shivering thermogenesis is mediated primarily by uncoupling protein 1 (UCP1), the development of the UCP1 knockout mouse has enabled the study of possible UCP1-independent non-shivering thermogenic mechanisms, whose existence has been shown so far only indirectly in white adipose tissue and still continues to be a matter of debate in BAT. In this study, by using magnetic resonance thermometry with hyperpolarized xenon, we produce the first direct evidence of UCP1-independent BAT thermogenesis in knockout mice. We found that, following adrenergic stimulation, the BAT temperature of knockout mice increases more and faster than rectal temperature. While with this study we cannot exclude or separate the physiological effect of norepinephrine on core body temperature, the fast increase of iBAT temperature seems to suggest the existence of a possible UCP1-independent thermogenic mechanism responsible for this temperature increase.

## Introduction

Brown adipose tissue (BAT) continues to spark interest in the biomedical research community as a potential target for the treatment of obesity and diabetes^1^. BAT is a tissue specialized in non-shivering thermogenesis (NST), a mechanism developed by mammals to defend core body temperature. In BAT, non-shivering thermogenesis is mediated primarily by the uncoupling protein 1 (UCP1). This protein uncouples fatty acid oxidation from adenosine triphosphate production, leading to a futile metabolic process that results in increased heat production^2^. As such, the presence of UCP1 in brown adipocytes has been thought of as a necessary condition for BAT thermogenesis.

One critical tool for studying non-shivering thermogenesis in BAT has been the development of UCP1 knockout (KO) mice^3^. This KO animal model has enabled studies aimed at identifying possible UCP1-independent thermogenic mechanisms. While initial work done in cold acclimated animals has clearly shown the absence of non-UCP1 mediated adaptive thermogenic mechanisms^4–6^, in other tissues, such as beige fat^7–9^, white adipose tissue^10^, and/or skeletal muscle mitochondria^11^, more recent studies have suggested that alternative, non-adaptive mechanisms of thermogenesis that are independent of UCP1 may exist. The major drawback of these studies is that UCP1-independent thermogenesis was assessed, in all cases, indirectly, by either measurements of oxygen consumption in a controlled environment^7,8,10^, fat oxidation in tissue suspensions^8,10,11^, and/or increased adenosine monophosphate-activated protein kinase activity in dissected tissues^10^.

The most direct way to determine whether non-adaptive, UCP1-independent thermogenic mechanisms exist in BAT would be via direct measurements of the temperature of this tissue in intact KO animals. However, non-invasive temperature measurements of tissues deep inside the body are notoriously very difficult. Optical techniques like near-infrared thermography^12,13^ have the advantage of being minimally invasive and cost-effective for large studies; however, these measurements assess BAT temperature only indirectly via superficial skin temperature measurements that do not have the necessary sensitivity and specificity to detect small temperature changes that may occur a few mm beneath the skin^14–18^. Furthermore, optical thermographic techniques are prone to artefacts from the environmental conditions under which these measurements are performed, even when a reference region is used for relative temperature measurements^19^. Wire thermistors^20^, thermocouples^8,21^, and fiber optic temperature probes^22^ have also been used. Even though the use of these probes is relatively straightforward and enables good temporal resolution, in our experience their measurement is extremely sensitive to probe positioning. In addition, the relatively large heat capacity of these probes may prevent the detection of small, but important, tissue temperature changes that could occur in the intact tissue^23^. It is also important to point out that these temperature probes cannot provide a ground truth for measurements of iBAT temperature, as these probes cannot be inserted directly into the iBAT depot without causing significant hemorrhage, which would inevitably alter the state of the tissue.

While X-ray computed tomography^24,25^ may offer another avenue to measure the temperature of tissues deep inside the body *in vivo*^26,27^ non-invasively, it may not provide the sensitivity necessary to detect small changes in BAT temperature^28–30^. More importantly, temperature-induced changes in tissue radiodensity in CT images cannot be decoupled from changes in tissue lipid content, also known to occur in BAT during NST.

Magnetic resonance imaging (MRI) offers another means for non-invasive, *in vivo* temperature measurements. Several MR relevant parameters are indeed temperature sensitive. Among all, the proton resonance frequency shift (PRF) of water molecules is the more appealing MR temperature probe. The water PRF is appealing because the temperature-induced frequency shift, −0.01 ppm/°C, is linear and almost independent of tissue type. As such, unlike other MR thermometry methods, PRF-based thermometry methods do not require pre-calibration in the tissue of interest. More interestingly, the water PRF can be used to measure absolute temperature, provided that a temperature-insensitive resonance frequency can be found. In the brain, for example, the N-acetyl aspartate protons have been used as a temperature-insensitive reference for the PRF to obtain information on absolute temperature^31–35^. In other parts of the body, the temperature-insensitive resonance frequency of methylene protons has been proposed as a possible reference to measure relative and absolute temperature in tissues containing fat. However, because at the microscopic level water and fat spins always reside in magnetically different compartments, these techniques are typically not very accurate^36–40^. Microscopic magnetic susceptibility gradients generated at water-fat interfaces affect water and fat frequencies differently, leading to apparent water-fat frequency shifts comparable to the expected temperature-induced frequency shift. These susceptibility induced frequency shifts strongly depend on the specific distribution of water and fat spins within a given voxel, precluding the possibility to measure absolute temperature, while degrading the accuracy of relative temperature measurements^41^. To further complicate the issue, local changes in tissue oxygenation, perfusion, and lipid content that are known to occur in BAT during NST are expected to lead to a much larger PRF shift that cannot be decoupled from the much smaller temperature-induced shift.

Recently, Branca *et al*. showed that the linear temperature dependence of the chemical shift of xenon dissolved in lipids (~−0.2 ppm/°C, *i.e*. 20 fold higher than that of water protons typically used for MR thermometry), coupled with the specific accumulation of xenon in BAT during stimulation of thermogenesis^42^, could be used to directly measure relative temperature changes in BAT with much higher accuracy^43^. This method was used to directly monitor BAT thermogenic activity in lean and obese mice^43^. More recently it was shown that, by referencing the temperature-dependent chemical shift of lipid-dissolved xenon to the temperature-independent chemical shift of nearby lipid protons, frequency shifts induced by macro- and microscopic susceptibility gradients could be fully removed to obtain absolute temperature information^36,44,45^. Here, absolute MR thermometry by lipid-dissolved, hyperpolarized ^129^Xe (XeMRT) is used to measure directly BAT temperature *in vivo* in UCP1−/− (knock out or KO) mice to assess whether BAT in KO mice is thermogenically competent.

## Results

For these studies, a colony of UCP1 KO and UCP1 +/+ (wild type or WT) mice was generated by a single breeding pair of UCP1 heterozygous mice with a C57BL/6 genetic background. All mice were genotyped by PCR of mouse tail DNA, while dissection and immunohistochemical staining of excised interscapular BAT (iBAT) was used to confirm UCP1 ablation.

**Figure 1:**
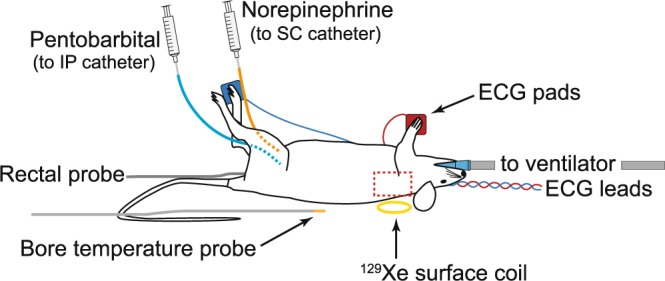
Experimental setup. The red dashed line indicates the anatomical region of interest which also coincides with the sensitive region of the ^129^Xe surface coil. Following pentobarbital anaesthesia, mice were intubated, mechanically ventilated, and placed on a small animal cradle. MR-compatible electrocardiogram (ECG) and rectal probes were used to monitor animal physiology during the experiment, while a second temperature probe was placed next to the animal to monitor bore temperature and provide a feedback to the forced-heated-air temperature controller. Pentobarbital and norepinephrine injections were administered *in situ* via intraperitoneal (IP) and subcutaneous (SC) catheters, respectively, attached to lines running out of the magnet bore to syringes.

For the thermometry experiments, mice were anesthetized with pentobarbital, one of the few anaesthetics known not to inhibit BAT thermogenesis and to have no effect on basal oxygen consumption or maximal norepinephrine-induced oxygen consumption^46^. After anaesthesia induction, mice were placed in the bore of a 9.4T MRI magnet equipped with a MR-compatible, closed-loop temperature control system, capable of maintaining a constant bore temperature within one tenth of a degree Celsius. Mouse rectal temperature was measured by a MR-compatible thermistor probe, while BAT temperature was measured by XeMRT (Fig. 1).

After acquisition of anatomical images, first order shimming gradients were used to correct for magnetic field inhomogeneities in a volume centred in the iBAT region from which ^1^H spectra, used to determine absolute BAT temperature via XeMRT, were acquired.

**Figure 2:**
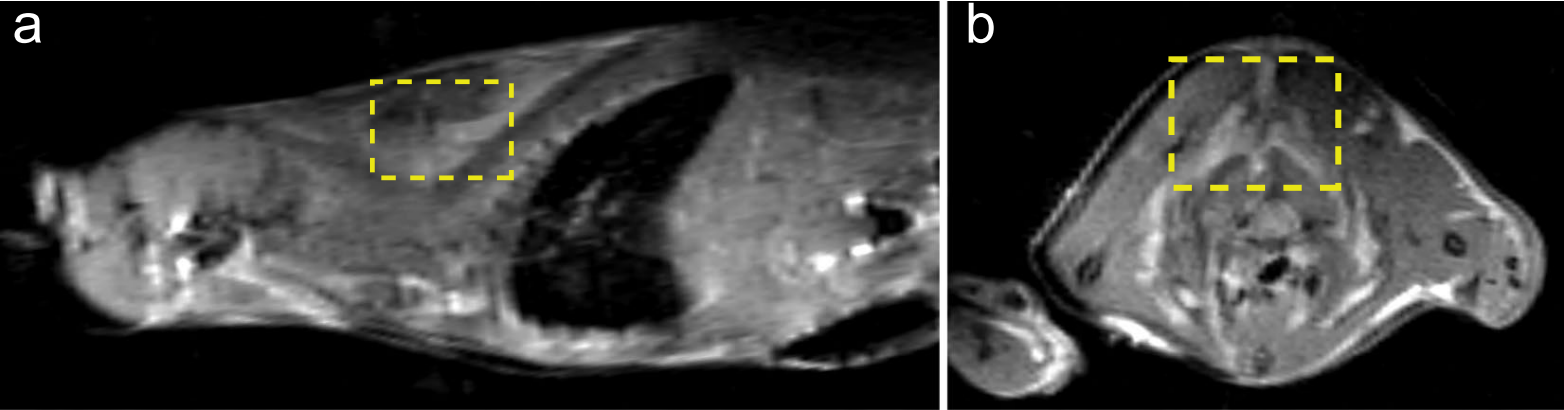
Anatomical ^1^H images showing the shimmed region from which localized spectra used to determine iBAT temperature were acquired. Representative sagittal (**a**) and axial (**b**) images of one KO mouse with the localized spectroscopy voxel outlined in yellow. The ^129^Xe surface coil was located right above this region.

Figure 2 shows an example of anatomical ^1^H images indicating the volume of interest, encompassing the iBAT depot (hyperintense regions in the figure), from which ^1^H spectra were acquired. A small ^129^Xe surface coil (10 mm diameter), located right beneath the iBAT, was used to collect non-localized xenon spectra used to measure and monitor iBAT temperature after norepinephrine injection. In some mice, sufficient signal was available to acquire ^129^Xe signal spectra using a localized spectroscopy sequence. Unlike the non-localized spectroscopy sequence, which collected the lipid-dissolved xenon signal from all interscapular brown adipose tissue located within the sensitive region of the surface coil (~1 cm^3^), the localized spectroscopy sequence spatially selected the ^129^Xe signal originating from the exact same voxel from which ^1^H spectra were acquired.

**Figure 3:**
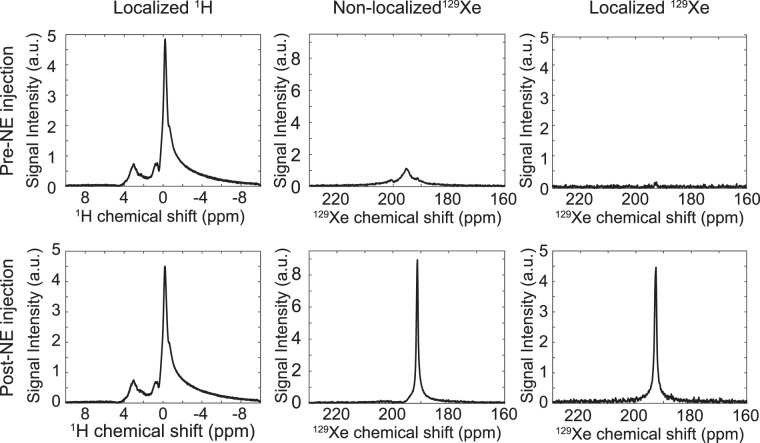
^1^H and ^129^Xe spectra from a KO animal before and after norepinephrine injection. No changes are observed in the localized ^1^H spectra before and after norepinephrine injection, whereas a large signal enhancement is observed in localized and non-localized ^129^Xe spectra right after norepinephrine injection as a result of iBAT activation and shunting of blood to this tissue^42^. ^1^H chemical shifts are referenced to the spectrometer centre frequency and ^129^Xe spectra are referenced to the methylene signal, as described in previous work^44,45^.

Figure 3 shows examples of ^1^H and ^129^Xe spectra acquired from a KO animal. Prior to norepinephrine injection, non-localized ^129^Xe spectra contain broad signal components originating from fat and non-fat tissues located within the sensitive region of the surface coil, encompassing iBAT, surrounding muscle, and white adipose tissue. In localized xenon spectra, only lipid-dissolved spin components (193 ppm peak) with sufficiently long transverse relaxation time, originating exclusively from the selected voxel, are detected. As expected, given the short repetition times used in these sequences (4 s), in both spectra the intensity of the lipid-dissolved ^129^Xe is too small to be quantified, preventing measurement of iBAT temperature before stimulation. However, right after stimulation of NST by norepinephrine, an average 30-fold enhancement in the lipid-dissolved ^129^Xe peak can be observed in the non-localized spectra as well as in the localized spectra. This enhancement, originating from the selective increase in blood flow to BAT^42^, enables clear identification of the lipid-dissolved ^129^Xe peak and measurement of its frequency from which iBAT temperature was derived. Given that, in the non-localized xenon spectra acquired before stimulation of NST, some of the signal originates from ^129^Xe dissolved in other tissues, the 30-fold enhancement seen in the post-stimulation spectra is clearly an underestimation of the actual signal enhancement that occurs as a result of the increased uptake of ^129^Xe in BAT^36,42,43,47,48^. The relative signal enhancement in the localized ^129^Xe spectra more closely reflects the actual specific enhancement in xenon uptake in iBAT; however, the low signal-to-noise ratio (SNR) associated with localized spectra acquired before stimulation prevented an accurate quantification of the lipid-dissolved ^129^Xe such that specific signal enhancement could not be directly quantified. On the other hand, no significant differences were noted between localized ^1^H spectra acquired before and after stimulation of NST.

**Figure 4:**
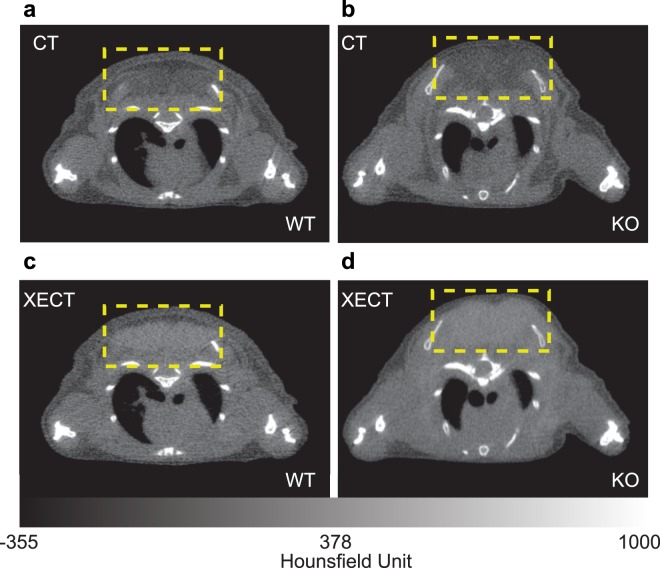
Selective radiodensity enhancement of iBAT after norepinephrine stimulation of NST in a WT and in a KO mouse. Axial (**a**) CT and (**b**) xenon-enhanced CT (XECT) images of a WT mouse after norepinephrine injection. Axial (**c**) CT and (**d**) XECT images of a KO mouse after norepinephrine injection. In both WT and KO mice adrenergic stimulation of BAT resulted in a radiodensity enhancement of BAT of more than 200 HU (yellow dashed box).

While ^129^Xe spectroscopy clearly indicates that the major xenon signal originates from xenon atoms that reside in lipid compartments, xenon-enhanced computed tomography (XECT) confirmed that these lipid compartments are the lipid compartments in iBAT. A selective radiodensity enhancement of over 200 Hounsfield units (HU) was observed in the iBAT of KO mice, similar to the enhancement observed in WT mice (Fig. 4) and also reported in previous work^42^. CT measurements of radiodensity enhancement in KO animals confirmed negligible xenon uptake in white adipose tissue, thus confirming that the lipid-dissolved xenon signal observed in ^129^Xe spectra after norepinephrine stimulation originates primarily from xenon dissolved in the lipid droplets of brown adipocytes.

**Figure 5:**
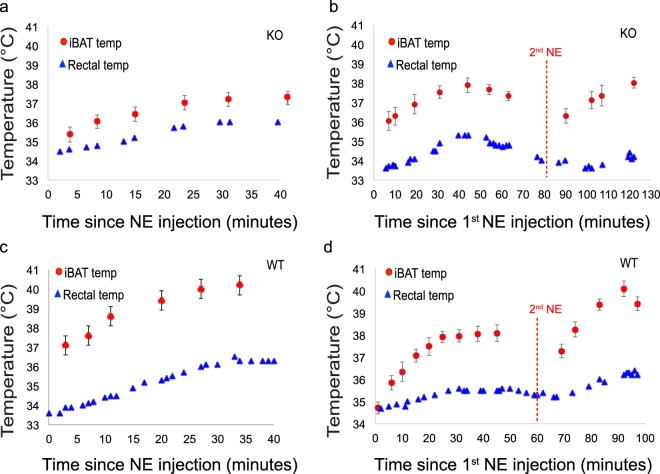
Rectal and iBAT temperature time curves following the first norepinephrine injection for two KO mice (**a**,**b**) and two WT mice (**c**,**d**). Rectal temperature (blue triangles) as well as iBAT temperature, as measured by XeMRT using a non-localized (red circles) ^129^Xe spectroscopy sequence (*i.e.* excitation followed by acquisition), as a function of time following the first norepinephrine injection. A second norepinephrine injection (red dashed line labelled “2^nd^ NE”) in both WT and KO was able to elicit a second thermogenic response in iBAT. Rectal temperature errors (±0.1 °C) are within the size of the data points.

Figure 5 shows examples of iBAT temperature measurements obtained from KO and WT mice right after stimulation of thermogenesis by norepinephrine, when the lipid-dissolved ^129^Xe frequency can be clearly identified and measured. In both mouse phenotypes, iBAT temperature increases steadily after norepinephrine injection for about 40–50 minutes, after which iBAT stimulation ceases and the lipid-dissolved ^129^Xe signal intensity returns to baseline levels, preventing further temperature measurements. In two mice (one WT and one KO), a second norepinephrine injection could be performed during the experimental time. In both cases, mice responded to the second norepinephrine injection with a further increase in iBAT temperature.

**Figure 6:**
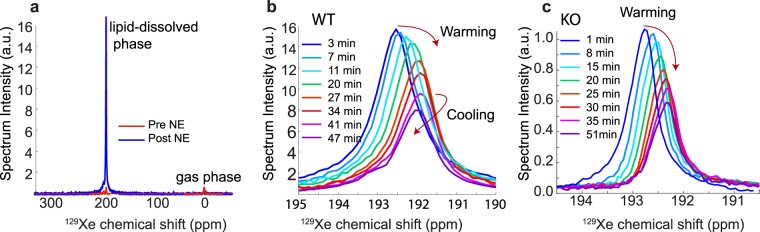
Example of lipid-dissolved ^129^Xe spectra acquired in WT and KO mice following adrenergic stimulation of iBAT. (**a**) Change in the lipid-dissolved xenon signal intensity before and after NE injection. Signal intensity is normalized by using the gas-phase peak, to correct for differences in ^129^Xe polarization between the two acquisitions. (**b**) Time series of spectra acquired from a WT mouse right after NE injection showing the temperature-induced frequency drift of the lipid-dissolved xenon peak for the same mouse shown in (**a**). (**c**) Time series of spectra acquired from a UCP1 KO mouse right after NE injection showing the temperature-induced frequency drift of the lipid-dissolved xenon peak. Please note that in (**b**,**c)** spectra amplitudes are scaled for clarity. All spectra were acquired using the non-localized sequence and corrected to remove fast-relaxing spin components originating from spins outside of the shimmed voxel.

**Figure 7:**
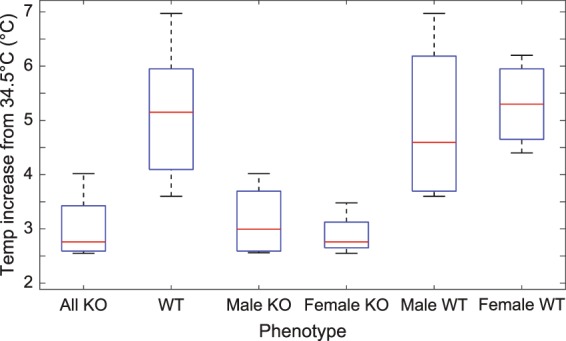
Box plots of maximum iBAT temperature increases above 34.5 °C for all animals as measured by XeMRT following norepinephrine injection. The mean ± standard deviation for each group of mice is as follows: (3.0 ± 0.5) °C for all n = 8 KO mice, (5.1 ± 1.2) °C for n = 8 WT mice, (3.1 ± 0.7) °C for n = 4 male KO mice, (4.9 ± 1.6) °C for n = 4 male WT mice, (2.9 ± 0.4) °C for n = 4 female KO mice, and (5.3 ± 0.8) °C for n = 4 female WT mice. The mean BAT temperature increase above 34.5 °C was significant in both WT (one-tailed *t* test, p < 3 × 10^−6^) and KO (one-tailed t test, p < 0.0001) groups. The difference in the mean BAT temperature increase between the WT and KO groups was also significant (two-sample *t* test, p < 0.0004, power = 0.995). No difference was observed between either male and female KO mice (two-sample *t* test, p = 0.55) or between male and female WT mice (two-sample *t* test, p = 0.70).

Figure 6 shows the remarkable temperature-induced drift of the lipid-dissolved ^129^Xe peak used to measure iBAT temperature changes. Although the increase in iBAT temperatures in WT mice was generally greater than in KO mice, in both phenotypes the increase in iBAT temperature was higher than the increase in rectal temperature, suggesting that heat was generated in or near iBAT. A summary of the maximum temperature increase above the equilibrated temperature of 34.5 °C observed in WT and KO mice is provided in the box plot in Fig. 7. The iBAT temperature increase above the equilibrated temperature was significant in both WT (one-tailed *t* test, p < 3 × 10^−6^, n = 8, t = 12.3, degrees of freedom = 7) and KO (one-tailed *t* test, p < 0.0001, n = 8, t = 15.7, degrees of freedom = 7) groups. Additionally, the mean iBAT temperature increase above the equilibrated temperature of 34.5 °C was significantly different between the WT and KO groups (two-sample *t* test, p < 0.0004, n = 16, t = 4.6, degrees of freedom = 14, power = 0.995). Upon norepinephrine injection, rectal temperature also increased. Specifically, we measured a mean maximum rectal temperature increase in both WT and KO mice of 2.09 ± 1.36 °C and 1.16 ± 0.72 °C, respectively. The difference in rectal temperature increase, however, was found not to be statistically different. Significance was in this case determined by using a two-sample *t* test, at a significance level of 5%, assuming equal variances. Variances were found to be equal using an F-test at the 5% significance level.

**Figure 8:**
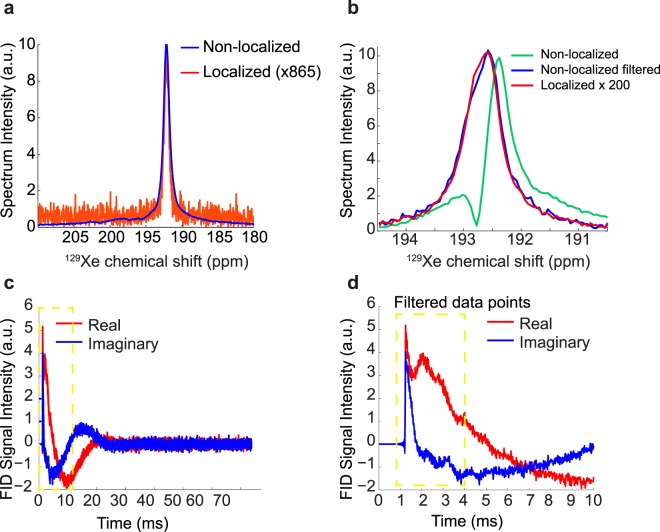
Comparison between non-localized and localized ^129^Xe spectra used to determine iBAT temperature in a KO mouse. **(a)** Non-localized ^129^Xe spectrum (blue) overlaid onto a localized ^129^Xe spectrum (red). A more than 500-fold reduction in SNR is seen in the localized spectra compared to the non-localized spectra. **(b)** Non-localized spectrum (green) distorted by lipid-dissolved xenon spin components located outside the shimmed volume. (blue) Same non-localized spectrum obtained after filtering out the fast-relaxing spin components. (red) Localized spectrum acquired on the same mouse. **(c)** Corresponding FID signal obtained with the non-localized spectroscopy sequence. Fast-relaxing spin components are observed only during the first few milliseconds of the FID’s evolution (yellow dotted box). **(d)** First 10 ms of the FID showing the contribution of fast relaxing spin components mainly in the first 4 ms (yellow dotted box).

While a clear signal enhancement was consistently observed in all mice using a non-localized spectroscopy sequence, localized spectra with sufficient SNR could be acquired only in a subset of the mice (3 KO mice). Localized ^129^Xe spectra are preferable to non-localized ^129^Xe spectra for accurate temperature measurements, as the ^129^Xe signal originates from the same region of the ^1^H signal used as reference. However, in our experiments, the longer echo time characteristic of these sequences and the use of non-adiabatic radiofrequency pulses, which inherently lead to a signal reduction when used in conjunction with surface coils, produced spectra with significantly lower SNR than non-localized spectra (Fig. 8). In two of the mice in which both localized and non-localized spectra were acquired, a discrepancy between localized and non-localized iBAT temperature measurements was found.

This apparent discrepancy was due to the mismatch between the shimmed voxel, from which the localized spectra were acquired, and the sensitive region of the coil from which the non-localized spectra were acquired. Specifically, the sensitive region of the coil included iBAT that was outside of the shimmed voxel. In these regions, the signal is subjected to field inhomogeneities, which lead to a faster transverse relaxation time and to distortion and broadening of the lipid-dissolved xenon spectral line (Fig. 8b, green spectrum). However, the contribution of these fast-relaxing spins, which can be seen in the early evolution of the free induction decay (FID) signal (Fig. 8d), can be completely eliminated by filtering out the early FID’s data points. Such a filtering procedure leads to non-localized spectra that nicely match the localized spectra (Fig. 8b red and blue spectra).

## Discussion

**Figure 9:**
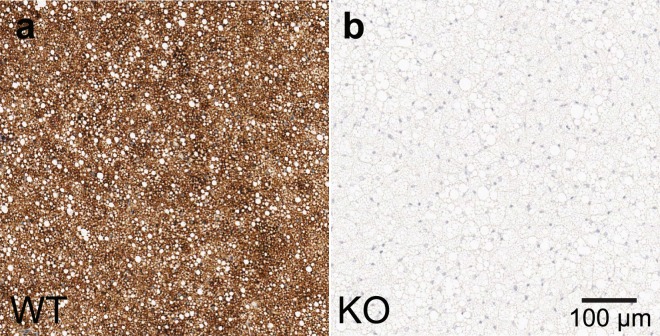
Immunohistochemistry staining for UCP1 of interscapular BAT dissected from WT and KO mice. Imaging validation of the (**a**) WT and (**b**) KO phenotypes from their respective breeding colonies.

In this study we present direct evidence of an increase in the iBAT temperature of KO mice after norepinephrine injection, suggesting that the iBAT of KO mice is thermogenically competent. Absolute iBAT temperature measurements by XeMRT show that iBAT temperature increases with norepinephrine stimulation (Fig. 7), despite ablation of UCP1 (Fig. 9). Specifically, in almost every KO mouse, the rate at which temperature increases is faster than rectal temperature. This strongly suggests that some form of non-shivering thermogenesis, independent of UCP1, also takes place in or very near the iBAT of KO mice. As such, these results seem to lend credence to previous investigations that have suggested the existence of other thermogenic mechanisms independent of UCP1^7–11,49^. Specifically, recent studies have presented evidence for three possible UCP1-independent thermogenic mechanisms in brown adipocytes^50^: genetic evidence for creatine-regulated substrate cycling^7,51^; genetic, physiological monitoring, and a series of *in vitro* assay evidence for calcium-dependent hydrolysis of adenosine triphosphate^8^; and core body temperature data for proton leak by uncoupling protein 3^52^. However, in these studies, the existence of UCP1-independent thermogenic mechanisms was demonstrated only indirectly. The current study, on the other hand, represents the first direct observation of UCP1-independent non-shivering thermogenesis in BAT in intact living animals.

Interestingly, while previous PET measurements utilizing ^18^F-fluorodeoxyglucose (FDG) uptake to quantify iBAT activation have shown a significant difference in FDG uptake between male and female KO mice^53^, this study shows no difference in iBAT thermogenic response between male and female KO mice. This is not surprising, as more recent studies have shown FDG uptake not to be a reliable indicator of thermogenesis in BAT^13^.

Similar iBAT temperature increase is observed in WT mice after NE injection. However, as expected from previous work, iBAT temperature increase after norepinephrine stimulation of NST is significantly higher in WT mice compared to KO mice.

While the XeMRT method can provide direct information on absolute tissue temperature, it has some limitations. Specifically, the major drawback of the proposed technique is that it is not widely available, since specialized hardware is required to produce hyperpolarized ^129^Xe gas and to detect the ^129^Xe signal.

The other limitation is the reduced spatial resolution. In this study, by performing a single spectroscopic measurement of the entire iBAT depot, we measured the average iBAT temperature. To improve spatial resolution one could use chemical shift encoding techniques, as previously shown^36,54^. However, with our current levels of ^129^Xe polarization, ranging from 10–16%, spatial resolution is still limited to about 0.5 cm^3^. While higher ^129^Xe polarizations could enable temperature mapping with higher spatial resolution, this would be at the expense of the temporal resolution, which is needed for studies in small animals to appreciate the temporal response of BAT to stimulation. Spatial resolution is needed in MR thermometry to either reduce partial volume effect or to detect possible temperature gradients across the tissue. However, it is important to notice that XeMRT thermometry of BAT does not suffer from partial volume effect. This is because more than 90% of the detected lipid-dissolved ^129^Xe signal originates from ^129^Xe dissolved in the lipid droplets of BAT. As such, the XeMRT temperature measurements still accurately reflect iBAT temperature, regardless of the spatial resolution used for the XeMRT measurement. The XECT images shown in Fig. 4 further illustrate the selective uptake of xenon in iBAT in both WT and KO animals during stimulation of thermogenesis, which gives rise to the remarkable lipid-dissolved xenon signal enhancement observed in the spectra of Fig. 6. The selective increase in xenon uptake in the iBAT of these animals is a direct consequence of the remarkable, selective increase in blood flow to this tissue that has been shown to occur in both WT and KO animals^55^, despite that, in the latter, thermogenic activity is considerably reduced. While temperature mapping is not expected to increase the accuracy of XeMRT, it could help detect possible temperature gradients across the iBAT depot. However, given the relatively low heterogeneity and high vascular perfusion of the iBAT depot in mice, it is highly unlikely that temperature gradients, even if present, would be strong enough to be detected.

It is important to note that, even though the lipid-dissolved xenon signal originates almost exclusively from xenon dissolved in iBAT after NE injection, when volume coils or relatively large surface coils are used, the presence of other BAT depots within the sensitive region of the coil, but outside the shimmed region from which the reference lipid-proton signal is detected, may degrade spectral resolution and the accuracy with which absolute temperature can be measured. In this case, fully adiabatic voxel-selective spectroscopy sequences, such as LASER (Localization by Adiabatic SElective Refocusing), should be used in conjunction with surface coils. These sequences, however, typically require a much better field homogeneity than the one we were able to obtain in iBAT, as the signal is typically acquired at much longer echo times. An alternative to the use of localized spectroscopic techniques is direct time-domain filtering of fast-relaxing spin components that originate from inhomogeneous iBAT regions located outside the shimmed volume, as shown in Fig. 8. By filtering out these fast-relaxing spin components, non-localized xenon spectra yield narrow spectral lines, similar to those obtained with localized spectroscopy sequences, but with a much higher spectral SNR.

Finally, while the XeMRT method reports directly on brown adipocyte temperature, it cannot identify the specific thermogenic mechanism at work. Notably, in this study we cannot exclude that the increase in iBAT temperature seen in UCP1 KO mice is, in part or as a whole, due to the systemic pharmacological effects of norepinephrine, which is also expected to play a role that cannot be decoupled from other possible thermogenic mechanisms. Indeed, in addition to a significant increase in heart rate, NE also causes vasoconstriction, which is expected to lead to a reduction in heat dissipation and to an increase in core body temperature.

In summary, this study shows a remarkable increase in the iBAT temperature of UCP1 KO mice following adrenergic stimulation of NST. Whether or not this is due to a systemic effect of NE, to a UCP1-independent thermogenic mechanism, or to a combination of both, will require further investigation. However, the fact that iBAT absolute temperature is significantly higher than rectal temperature is strongly suggestive of a UCP1-independent contribution. As such, these measurements provide the first direct evidence of a possible UCP1-independent, non-shivering thermogenesis mechanism in the iBAT of UCP1 KO mice.

## Methods

### Animal protocol

All animal experiments were performed according to the ethical guidelines for animal experiments as described in the Public Health Service Policy on Humane Care and Use of Laboratory Animals^56^, the Animal Welfare Act and Animal Welfare Regulations^57^ and the Guide for the Care and Use of Laboratory Animals^58^ under an animal protocol approved by the Institutional Animal Care and Use Committee of the University of North Carolina at Chapel Hill. For these studies, a colony of UCP1+/+ (wild type or WT) mice and UCP1−/− (knock out or KO) mice was generated from a single breeding pair of UCP1 heterozygous mice with a C57BL/6 genetic background purchased from Jackson Laboratory. All mice were genotyped by PCR of mouse tail DNA, while immunochemistry of dissected interscapular BAT post-mortem was used to confirm mouse genotype (Fig. 9). The mouse colony was housed at 24 °C and fed a standard chow diet *ad libitum* until imaging.

For the MR temperature experiment, a total of 8 WT mice (4 male, 4 female) and 8 KO mice (4 male, 4 female) were used. For these studies, each mouse was anesthetized with an intraperitoneal injection of pentobarbital (Oak Pharmaceuticals, Lake Forest, Illinois, USA) at a dose of 70 mg/kg, followed by tracheal intubation. An intraperitoneal catheter was placed to provide maintenance doses of pentobarbital (¼ of the initial dose every 40 minutes), while a subcutaneous catheter was placed for norepinephrine injection (Levophed norepinephrine bitartrate, Hospira, Inc., Lake Forest, Illinois, USA) at a dose of 1 mg/kg.

All imaging experiments were performed on a 9.4 T spectrometer (Bruker BioSpec 94/30, Bruker Biospin Corp., Billerica, Massachusetts, USA). With the exception of the first male WT mouse, in which all temperature measurements were made by using a 35 mm dual-tuned ^1^H/^129^Xe volume quadrature coil (m2m Imaging Corp.), to increase sensitivity, all XeMRT measurements were made by using a 10 mm ^129^Xe surface coil (m2m Imaging Corp.) placed inside a 72 mm ^1^H quadrature volume coil (Bruker Biospin Corp.). Care was used to place the anesthetized mouse supine, with the sensitive region of the ^129^Xe surface coil located right under the interscapular BAT depot.

During the experiment, mice were mechanically ventilated using a homemade ventilator, similar to one described in previous work^59^, at a rate of 80 breaths per minute and with a tidal volume of 0.15 mL of a 30-vol% O_2_ and 70-vol% N_2_ gas mixture. For the XeMRT measurements, the N_2_ volume was replaced with an equivalent volume of hyperpolarized ^129^Xe gas with a polarization ranging between 10–16%. Throughout the experiment, mouse heart rate was monitored with a MR-compatible physiological monitoring system (Small Animal Instruments, Stony Brook, New York, USA). The spectrometer bore temperature was monitored by a MR-compatible thermocouple and maintained at 34 °C ± 1 °C by a forced-air heating system (Small Animal Instruments). Mouse rectal temperature was monitored via a MR-compatible rectal probe (Temp 9500, Oakton Instruments, Vernon Hills, Illinois, USA). Once in place, anesthetized mice were equilibrated to bore temperature for about 30 minutes. During this time, anatomical images were acquired and a volume of interest that included most of the interscapular BAT depot was shimmed for field homogeneity, as described below. After 30 minutes from the beginning of the experiment, mouse rectal temperature consistently stabilized around 34.5 °C. Once equilibrated, periodic BAT and rectal temperature measurements were made using XeMRT and the rectal temperature probe, respectively. After the acquisition of 2–3 temperature data points, mice were injected with norepinephrine at a dose of 1 mg/kg. After norepinephrine injection, a series of XeMRT temperature measurements were performed every 3–10 minutes for the entire duration of BAT stimulation, which lasted up to about 40–50 minutes from the first norepinephrine injection. In two animals, a second norepinephrine injection was performed about one hour from the first injection. At the end of the MR experiment, mice were euthanized with an overdose of pentobarbital.

### *In vivo*^129^Xe magnetic resonance thermometry

All MR scans were respiratory triggered to reduce motion artefacts. Anatomical axial ^1^H scans were acquired with a spin echo sequence using a 30 × 30 mm^2^ field of view, 256 × 128 matrix, 6 slices, 2 mm slice thickness, 5.58 ms echo time, 1 s repetition time, and 1 average. Volumes of interest containing primarily interscapular BAT were identified in the anatomical images and the signal selected from a localized point-resolved spectroscopy sequence (PRESS) was manually shimmed with first order shimming gradients using the ^1^H methylene peak. The localized ^1^H spectra from the same region were acquired immediately before and after the acquisition of ^129^Xe spectra with 2048 complex points, 20 ppm bandwidth, 12 averages, 2 s repetition time, 18.2 ms echo time, and 400.321 MHz basic frequency. ^129^Xe spectra were acquired with a non-localized sequence (90 °- acquisition sequence, with 4096 complex points, 500 ppm bandwidth, 110.7518 MHz basic frequency, 4 s repetition time, and 15 averages, resulting in a total acquisition time of about 60 s) and, in 3 mice, also with a localized stimulated echo acquisition mode sequence (STEAM, 4096 complex points, 500 ppm bandwidth, 110.7518 MHz basic frequency, 6 ms echo time, 7 ms mixing time, 3.5 s repetition time, and 20 averages, resulting in a total acquisition time of approximately 70 s).

Total acquisition time for a single XeMRT temperature measurement using the non-localized spectroscopy sequence and the localized spectroscopy sequence was 120 s and 130 s, respectively.

### Data analysis

XeMRT absolute temperature values were obtained by measuring the lipid-dissolved xenon frequency with respect to the resonance frequency of methylene protons, using the previously determined temperature calibration^45^:

1 $$T=\frac{{\delta }_{C{H}_{2}-ref}-(200.15\pm 0.03)\,ppm}{-(0.0058\pm .0010)\,ppm/^\circ C},$$

where ${\delta }_{C{H}_{2}-ref}$ is the methylene-referenced ^129^Xe (lipid-dissolved ^129^Xe) chemical shift. As detailed in previous work^44^, this shift is calculated with respect to a fictitious 0 ppm Xe reference derived from the resonance frequency of methylene protons, as measured in ^1^H spectra.

In regions of strong magnetic susceptibility gradients, such as iBAT, ill-defined peak shapes can result from the convolution of macro- and microscopic magnetic susceptibility and shimming gradients. As a result, typical post-processing procedures such as phasing, baseline correction, and peak-fitting of standard spectroscopic line shapes (e.g. Lorentzian, Lorentzian/Gaussian, Voigt) fail to reliably estimate the peak centroid and uncertainty. Therefore, two independent analyses were performed whereby ^1^H and ^129^Xe peak centroids were manually determined by two independent investigators from magnitude spectra. The average of the two peak centroids was then used to calculate the temperature, and half of the difference between the two values was used to determine the uncertainty in the temperature measurement.

### Statistical analyses

Group results are reported as mean ± standard deviation. Analyses were performed with a one-sided Student’s *t* test for significant BAT temperature change and a two-sample Student’s *t* test for group comparisons as implemented in MATLAB R2017b software (MathWorks, Natick, Massachusetts, USA). Values of p < 0.03 were considered significant.

### Immunohistochemistry analysis of BAT tissue

Mouse phenotypes were confirmed by immunohistochemistry analysis of excised BAT. For this analysis, after pentobarbital euthanasia, iBAT was dissected and fixed overnight in 4% paraformaldehyde at 4 °C. Samples were then dehydrated, cleared, embedded in paraffin, and sectioned into 4 μm slices. Sections were then dewaxed and rehydrated. ﻿Cyto-Q Background Buster (NB306; In-novex) was used for the blocking procedure, followed by UCP1 primary antibody incubation at room temperature (1:1000; catalogue no. ab10983; Abcam). Secondary antibody incubation was performed with biotinylated goat anti-rabbit (1:1000, BA100, Vector) and detection performed with Vectastain Elite ABC complex (Vector).

### Xenon-enhanced computed tomography

To assess directly the degree of xenon uptake in the adrenergically-stimulated BAT of both WT and KO mice, xenon-enhanced computed tomography scans were performed in six additional mice (three male WT mice and three male KO mice from the same colony) as previously described^42^. Images were acquired using a small animal CT scanner (GE eXplore speCTZ/CT, General Electric Company, Waukesha, Wisconsin, USA) with a tube peak voltage of 70 kVp, a current of 50 mA, 220 views, and a resolution of 100 µm. For these studies, mice were first anesthetized and then intubated as described above. Mice were mechanically ventilated at a rate of 80 breaths/minute with a tidal volume of 0.25 mL of a gas mixture containing 30-vol% O_2_ and 70-vol% N_2_. Non-enhanced CT images were acquired 10 minutes after a subcutaneous injection of norepinephrine at a dose of 1 mg/kg; xenon-enhanced CT images were acquired right after replacing N_2_ gas in the breathing gas mixture with an equivalent volume of non-radioactive xenon gas. CT image analysis was performed using VivoQuant software (Invicro, LLC, Boston, Massachusetts, USA).

## Data Availability

The datasets generated during the current study are available from the corresponding author on reasonable request.

## References

[1] Luo L, Liu M (2016). Adipose tissue in control of metabolism. J. Endocrinol..

[2] Cannon B, Nedergaard J (2004). Brown adipose tissue: function and physiological significance. Physiol. Rev..

[3] Enerbäck S (1997). Mice lacking mitochondrial uncoupling protein are cold-sensitive but not obese. Nature.

[4] Nedergaard J (2001). UCP1: The only protein able to mediate adaptive non-shivering thermogenesis and metabolic inefficiency. Biochimica et Biophysica Acta - Bioenergetics.

[5] Golozoubova V (2002). Only UCP1 can mediate adaptive nonshivering thermogenesis in the cold. FASEB J..

[6] Golozoubova V, Cannon B, Nedergaard J (2006). UCP1 is essential for adaptive adrenergic nonshivering thermogenesis. Am. J. Physiol. Metab..

[7] Kazak L (2015). A Creatine-Driven Substrate Cycle Enhances Energy Expenditure and Thermogenesis in Beige Fat. Cell.

[8] Ikeda K (2017). UCP1-independent signaling involving SERCA2b-mediated calcium cycling regulates beige fat thermogenesis and systemic glucose homeostasis. Nat. Med..

[9] Bradley CA (2017). Adipose tissue: Noncanonical beige fat thermogenesis. Nat. Rev. Endocrinol..

[10] Ukropec J, Anunciado RP, Ravussin Y, Hulver MW, Kozak LP (2006). UCP1-independent Thermogenesis in White Adipose Tissue of Cold-acclimated UCP1−/− Mice. J. Biol. Chem..

[11] Shabalina IG (2010). Cold tolerance of UCP1-ablated mice: A skeletal muscle mitochondria switch toward lipid oxidation with marked UCP3 up-regulation not associated with increased basal, fatty acid- or ROS-induced uncoupling or enhanced GDP effects. Biochim. Biophys. Acta - Bioenerg..

[12] Crane JD, Mottillo EP, Farncombe TH, Morrison KM, Steinberg GR (2014). A standardized infrared imaging technique that specifically detects UCP1-mediated thermogenesis *in vivo*. Mol. Metab..

[13] Hankir MK (2017). Dissociation Between Brown Adipose Tissue 18 F-FDG Uptake and Thermogenesis in Uncoupling Protein 1–Deficient Mice. J. Nucl. Med..

[14] El Hadi H (2016). Infrared thermography for indirect assessment of activation of brown adipose tissue in lean and obese male subjects. Physiol. Meas..

[15] Gatidis S (2016). Is It Possible to Detect Activated Brown Adipose Tissue in Humans Using Single-Time-Point Infrared Thermography under Thermoneutral Conditions? Impact of BMI and Subcutaneous Adipose Tissue Thickness. PLoS One.

[16] Haq T (2017). Optimizing the methodology for measuring supraclavicular skin temperature using infrared thermography; implications for measuring brown adipose tissue activity in humans. Sci. Rep..

[17] Jang, C. *et al*. Infrared thermography in the detection of brown adipose tissue in humans. *Physiol. Rep*. **2** (2014). 10.14814/phy2.12167 PMC4255799 25413316

[18] Ang QY (2017). A new method of infrared thermography for quantification of brown adipose tissue activation in healthy adults (TACTICAL): a randomized trial. J. Physiol. Sci..

[19] Law J (2018). Thermal Imaging Is a Noninvasive Alternative to PET/CT for Measurement of Brown Adipose Tissue Activity in Humans. J. Nucl. Med..

[20] Inokuma, K. *et al*. Uncoupling protein 1 is necessary for norepinephrine-induced glucose utilization in brown adipose tissue. *Diabetes*, 10.1038/nm.2297 (2005). 10.2337/diabetes.54.5.1385 15855324

[21] Jeong JH, Chang JS, Jo YH (2018). Intracellular glycolysis in brown adipose tissue is essential for optogenetically induced nonshivering thermogenesis in mice. Sci. Rep..

[22] Khanna A, Branca RT (2012). Detecting brown adipose tissue activity with BOLD MRI in mice. Magn. Reson. Med..

[23] Meyer CW, Ootsuka Y, Romanovsky AA (2017). Body Temperature Measurements for Metabolic Phenotyping in Mice. Front. Physiol..

[24] Bydder GM, Kreel L (1979). The temperature dependence of computed tomography attenuation values. J. Comput. Assist. Tomogr..

[25] Fallone BG, Moran PR, Podgorsak EB (1982). Noninvasive thermometry with a clinical x-ray CT scanner. Med. Phys..

[26] DeStefano, Z. *et al*. CT thermometry for cone-beam CT guided ablation. In *Medical Imaging 2016: Image-Guided Procedures, Robotic Interventions, and Modeling* (eds. Webster, R. J. & Yaniv, Z. R.) 9786, 978615 (2016).

[27] Mahnken AH, Bruners P (2011). CT thermometry: Will it ever become ready for use?. Int. J. Clin. Pract..

[28] Pandeya GD (2011). Feasibility of computed tomography based thermometry during interstitial laser heating in bovine liver. Eur. Radiol..

[29] Schena, E. *et al*. Feasibility assessment of CT-based thermometry for temperature monitoring during thermal procedure: Influence of ROI size and scan setting on metrological properties. In *2015 37th Annual International Conference of the IEEE Engineering in Medicine and Biology Society (EMBC)* 2015–Novem, 7893–7896 (IEEE, 2015). 10.1109/EMBC.2015.7320222 26738122

[30] Tan D (2019). Experimental assessment on feasibility of computed tomography-based thermometry for radiofrequency ablation on tissue equivalent polyacrylamide phantom. Int. J. Hyperth..

[31] Cady EB, D’Souza PC, Penrice J, Lorek A (1995). The Estimation of Local Brain Temperature by *in Vivo* 1H Magnetic Resonance Spectroscopy. Magn. Reson. Med..

[32] Bainbridge A (2013). Regional neonatal brain absolute thermometry by 1H MRS. NMR Biomed..

[33] Sone D (2017). Noninvasive detection of focal brain hyperthermia related to continuous epileptic activities using proton MR spectroscopy. Epilepsy Res..

[34] Mintzopoulos D, Ratai E-M, He J, Gonzalez RG, Kaufman MJ (2019). Simian immunodeficiency virus transiently increases brain temperature in rhesus monkeys: detection with magnetic resonance spectroscopy thermometry. Magn. Reson. Med..

[35] Zhu M, Bashir A, Ackerman JJ, Yablonskiy DA (2008). Improved calibration technique for *in vivo* proton MRS thermometry for brain temperature measurement. Magn. Reson. Med..

[36] Zhang L (2017). Accurate MR thermometry by hyperpolarized 129Xe. Magn. Reson. Med..

[37] Babourina-Brooks B (2015). MRS thermometry calibration at 3T: Effects of protein, ionic concentration and magnetic field strength. NMR Biomed..

[38] Poorter JD (1995). Noninvasive MRI thermometry with the proton resonance frequency method: Study of susceptibility effects. Magn. Reson. Med..

[39] Baron, P. *et al*. Influence of water and fat heterogeneity on fat-referenced MR thermometry. *Magnetic Resonance in Medicine*, 10.1002/mrm.25727 (2015). 10.1002/mrm.25727 25940426

[40] Dewal RP, Yang QX (2016). Volume of interest-based fourier transform method for calculation of static magnetic field maps from susceptibility distributions. Magn. Reson. Med..

[41] Sprinkhuizen SM (2010). Temperature-induced tissue susceptibility changes lead to significant temperature errors in PRFS-based MR thermometry during thermal interventions. Magn. Reson. Med..

[42] Branca RT (2018). Accurate quantification of brown adipose tissue mass by xenon-enhanced computed tomography. Proc. Natl. Acad. Sci..

[43] Branca RT (2014). Detection of brown adipose tissue and thermogenic activity in mice by hyperpolarized xenon MRI. Proc. Natl. Acad. Sci..

[44] Antonacci MA, Zhang L, Burant, McCallister D, Branca RT (2017). Simple and robust referencing system enables identification of dissolved-phase xenon spectral frequencies. Magn. Reson. Med..

[45] Antonacci MA, Zhang L, Degan S, Erdmann D, Branca RT (2019). Calibration of methylene-referenced lipid-dissolved xenon frequency for absolute MR temperature measurements. Magn. Reson. Med..

[46] Ohlson KBE, Lindahl SGE, Cannon B, Nedergaard J (2003). Thermogenesis inhibition in brown adipocytes is a specific property of volatile anesthetics. Anesthesiology.

[47] Branca, R. T. *et al*. Measurements of human brown adipose tissue temperature during cold exposure by hyperpolarized xenon MR thermometry. in *Proceedings of the International Society of Magnetic Resonance in Medicine* **968** (2018).

[48] Branca, R. T., Zhang, L., Burant, A., Katz, L. & McCallister, A. Detection of human brown adipose tissue by MRI with hyperpolarized Xe-129 gas and validation by FDG-PET/MRI. in *International Society of Magnetic Resonance in Medicine* **1054** (2016).

[49] Olsen JM (2017). β3-Adrenergically induced glucose uptake in brown adipose tissue is independent of UCP1 presence or activity: Mediation through the mTOR pathway. Mol. Metab..

[50] Chouchani ET, Kazak L, Spiegelman BM (2019). New Advances in Adaptive Thermogenesis: UCP1 and Beyond. Cell Metab..

[51] Kazak L (2017). Genetic Depletion of Adipocyte Creatine Metabolism Inhibits Diet-Induced Thermogenesis and Drives Obesity. Cell Metab..

[52] Riley CL (2016). The complementary and divergent roles of uncoupling proteins 1 and 3 in thermoregulation. J. Physiol..

[53] Jeanguillaume C (2013). Visualization of Activated BAT in Mice, with FDG-PET and Its Relation to UCP1. Adv. Mol. Imaging.

[54] Barskiy, D. A. *et al*. NMR Hyperpolarization Techniques of Gases. *Chem. - A Eur. J*. **23** (2017). 10.1002/chem.201603884 PMC5462469 27711999

[55] Baron, D. M. *et al*. *In vivo noninvasive characterization of brown adipose tissue blood flow by contrast ultrasound in mice*. **5** (2012). 10.1161/CIRCIMAGING.112.975607 PMC3471995 22776888

[56] Public Health Service Policy on Humane Care and Use of Laboratory Animals.

[57] United States Department of Agriculture Animal Welfare Act and Animal Welfare Regulations.

[58] *Guide for the Care and Use of Laboratory Animals*. (National Academies Press), 10.17226/12910 (2011). 21595115

[59] Nouls J, Fanarjian M, Hedlund L, Driehuys B (2011). A Constant-Volume Ventilator and Gas Recapture System for Hyperpolarized Gas MRI of Mouse and Rat Lungs. Concepts Magn. Reson. Part B. Magn. Reson. Eng..

